# Competitiveness Among Physicians in Japan: A Nationwide Cross-Sectional Study

**DOI:** 10.7759/cureus.104089

**Published:** 2026-02-22

**Authors:** Kota Sakaguchi, Haruka Kobayashi, Tomoe Otsuka, Yasuhisa Nakano, Daisaku Yamasaki, Yasuharu Tokuda, Takashi Watari

**Affiliations:** 1 General Medicine Center, Shimane University Hospital, Izumo, JPN; 2 Digestive and General Surgery, Misawa Air Base Hospital, Misawa, JPN; 3 Clinical Training Center, Kariya Toyota General Hospital, Kariya, JPN; 4 Diagnostic and Generalist Medicine, Dokkyo Medical University, Mibu, JPN; 5 Medical Healthcare Media Unit, Nikkei Business Publications, Tokyo, JPN; 6 General Medicine, Muribushi Okinawa Center for Teaching Hospitals, Okinawa, JPN; 7 Integrated Clinical Education Center, Kyoto University Hospital, Kyoto, JPN

**Keywords:** burnout, career development, competitiveness, medical specialties, physicians, professional culture, socioeconomic factors

## Abstract

Objectives

Competitiveness is a pivotal psychological construct driving motivation and professional development in medicine. While it can foster lifelong learning, maladaptive competitiveness is linked to physician burnout. Despite its significance, empirical evidence on the competitiveness of physicians in non-Western medical systems remains scarce. This study aimed to examine the distribution of competitiveness across medical specialties and its association with demographic, professional, and socioeconomic factors in a large nationwide cohort of Japanese physicians.

Materials and methods

We conducted a nationwide cross-sectional study in June 2022 using an online survey platform. A total of 5,441 practicing physicians (median age: 48 years) from various specialties and practice settings were included. Competitiveness was assessed using a validated 20-item scale (possible range: 20-140). Multivariable linear regression analysis was performed to identify independent factors associated with competitiveness scores after adjusting for age, gender, medical specialty, workplace characteristics, and socioeconomic background.

Results

The mean total competitiveness score was 58.79 (SD: 10.52). Scores differed significantly across specialties: junior residents exhibited the highest scores (M=62.17), followed by plastic surgeons (M=61.94), while psychiatrists showed the lowest (M=57.03) score. Multivariable analysis confirmed that junior residents (β=1.65; 95% CI: 0.06-3.24; p=0.042) had significantly higher scores, while psychiatrists (β=-1.68; 95% CI: -2.83 to -0.51; p=0.005) had lower scores compared to internal medicine practitioners. Other independent correlates of higher competitiveness included older age (β=0.05; p<0.001), male gender (β=2.37; p<0.001), working in large hospitals (≥300 beds: β=1.02; p=0.011), having children (β=0.98; p=0.006), and higher parental household income (β=1.11; p=0.003).

Conclusions

Physician competitiveness in Japan is a multidimensional construct influenced by career stage, professional culture, and personal background. Although differences across specialties are statistically significant, the overall variation and effect sizes are relatively modest. Given the cross-sectional design, these findings suggest that competitiveness is a widely observed but potentially dynamic characteristic, rather than a fixed or stable professional trait. These findings provide foundational insights for medical educators and policymakers to design career support systems that balance professional excellence with psychological well-being.

## Introduction

Competitiveness is a multifaceted psychological construct involving the tendency to compare one's performance with others [1], which significantly influences motivation and behavior [2]. It comprises two main dimensions: a "hypercompetitive attitude" driven by a desire to defeat others, often causing interpersonal stress [3], and a "personal development competitive attitude" that frames competition as an opportunity for self-improvement [4]. In professional domains, adaptive competitiveness drives motivation, whereas maladaptive forms can lead to burnout or unethical behavior [5].

In medicine, physicians face constant pressure to update their knowledge amidst rapid technological advancements [6], making lifelong learning a core element of professionalism [7]. Alongside intrinsic motivation [8], a healthy competitive attitude can drive this self-improvement by encouraging autonomous learning and the recognition of skill gaps [9]. However, despite its impact on professional development and well-being, empirical research on physician competitiveness remains scarce, particularly in non-Western contexts [10].

In Japan, the recent restructuring of the medical board certification system has introduced new career dynamics [11,12]. Furthermore, medical specialties differ significantly in required skills, organizational culture, and personality traits; for example, surgical fields prioritize rapid decision-making, whereas psychiatry emphasizes long-term empathy [13-16]. Whether competitiveness profiles vary across these distinct professional cultures merits investigation but has not been sufficiently examined nationwide.

Therefore, this study aimed to examine the distribution of competitiveness and its associated factors among Japanese physicians. Using a large-scale nationwide survey, we investigated how competitiveness correlates with medical specialty, as well as demographic and socioeconomic factors. Elucidating these associations provides foundational insights for designing career support systems that foster healthy professional growth.

## Materials and methods

Study design and participants

This nationwide cross-sectional study was conducted from June 1 to June 16, 2022, targeting physicians in Japan via Nikkei Medical Online, a web-based panel that includes physicians from diverse practice settings across the country. The survey was accessible to 200,299 registered physician members of the platform to ensure a broad representation of the physician population. Initial responses were obtained from 6,651 participants. After applying the exclusion criteria detailed below, a final analytical sample of 5,441 valid responses was obtained, yielding an estimated response rate of 2.7% (5,441/200,299).

Inclusion and exclusion criteria

Rigorous exclusion criteria were applied to the initial pool of respondents to ensure data quality. Responses were excluded if they met any of the following conditions: (1) missing data for one or more survey items; (2) self-identification as a student (medical or nursing) rather than a licensed physician; or (3) responses deemed inappropriate or invalid. Inappropriate responses were defined a priori as (a) selecting the same option for six or more consecutive items or (b) a total score of less than 50 on the Competitiveness Scale. This <50 cut-off was explicitly established to eliminate potential cases of careless responding or "straight-lining" (e.g., uniformly selecting the lowest possible options without properly reading the questions), thereby ensuring the overall quality and validity of the dataset.

Measures

Competitiveness Scale

Competitiveness was assessed using the Competitiveness Scale developed by Sekiguchi [1]. This 20-item scale comprises four sub-factors: "Appeal of Victory" (5 items), "Primacy of Winning" (5 items), "Challenge Orientation" (5 items), and "Desire for Evaluation by Others" (5 items). Each item is rated on a 7-point Likert scale ranging from 1 (strongly disagree) to 7 (strongly agree). The total competitiveness score is calculated as the sum of all 20 items, with a possible range of 20-140 points. Higher scores indicate higher competitiveness. The scale demonstrated sufficient internal consistency in this study (Cronbach's α=0.74).

Other Variables

Demographic and background variables included gender, age, and years since graduation. Regarding job-related characteristics, participants reported their primary medical specialty, leadership position status (e.g., department head, division chief), experience in rural or remote island medicine, bed capacity of their primary workplace (categorized into groups ranging from clinics to large hospitals), and the type of facility where they completed initial postgraduate training. Socioeconomic background was assessed using data on parental status (presence of children), living arrangements (living with/near parents), parents' profession (healthcare/non-healthcare), parental household income, and educational background (university type and admission method). Current residence was classified as "urban" or "rural".

Statistical analysis

All statistical analyses were performed using Stata SE version 17.0 (StataCorp, College Station, Texas, United States). Descriptive statistics were summarized as frequencies (n) and percentages (%) for categorical variables and as medians with interquartile ranges (IQR) or means with standard deviations (SD) for continuous variables, depending on the data distribution. Normality was assessed using the Shapiro-Wilk test. Group comparisons were conducted using the chi-squared test, t-test, Mann-Whitney U test, one-way ANOVA, or Kruskal-Wallis test, as appropriate. Multivariable linear regression analysis was performed to identify factors independently associated with total competitiveness scores. Explanatory variables were selected based on theoretical considerations and prior literature. For this analysis, "Junior Resident" was treated as a distinct category within the specialty variable to examine the specific characteristics of physicians in this early career phase. Internal Medicine served as the reference category. Statistical significance was defined as p<0.05. In addition to significance testing, regression coefficients (β) and 95% confidence intervals (CI) were evaluated to assess the associations.

Ethical considerations

This study was conducted in accordance with the Declaration of Helsinki and was approved by the Institutional Review Board of Shimane University (approval number: 20211124-1). Although the IRB protocol was titled "Comparison of empathy between doctors and nurses in Japan", the present study on competitiveness was conducted as a component of this approved project.

## Results

Participant selection and characteristics

**Figure 1:**
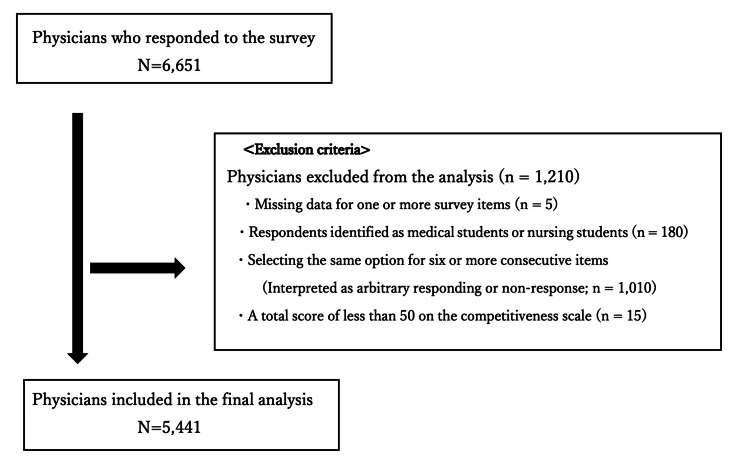
Flowchart of participant selection The initial pool consisted of 6,651 physicians who responded to the survey. After applying exclusion criteria (missing data, students, inappropriate responses), the final analytical sample comprised 5,441 physicians.

A total of 6,651 physicians initially responded to the survey. Based on the pre-defined exclusion criteria, 1,210 respondents were excluded. The specific breakdown of exclusions was as follows: missing data (n=5), self-identification as a medical or nursing student (n=180), and inappropriate responses (n=1,025), including straight-lining (n=1,010) or total scores below the cut-off (n=15). Consequently, the final analytical sample comprised 5,441 physicians (Figure 1).

The demographic and professional characteristics of the participants are summarized in Table 1. The median age was 48 years (IQR: 39.0-57.0), and 83.5% (n=4,545) were male. In terms of the practice setting, the sample consisted predominantly of hospital-based physicians (74.8% (n=4,069) working at facilities with ≥20 beds); however, 20.7% (n=1,126) were practicing in clinic settings (≤19 beds). Furthermore, 65.8% of respondents held a leadership position, and 18.5% reported experience in rural medicine.

**Table 1:** Participant characteristics (N=5,441) IQR: interquartile range; JPY: Japanese yen

Variable	n (%)
Demographic data	
Age, median (IQR)	48 (38-59)
Years after graduation, median (IQR)	22 (12-32)
Gender	
Male	4,543 (83.50%)
Female	864 (15.88%)
Other	34 (0.62%)
Job characteristics	
Leadership position	
Yes	3,578 (65.76%)
No	1,863 (34.24%)
Engaged in rural healthcare	
Yes	1,004 (18.45%)
No	4,437 (81.55%)
Number of beds where they work	
≤19	1,126 (20.69%)
20-99	301 (5.53%)
100-299	1,206 (22.17%)
≥300	2,562 (47.09%)
Other	246 (4.52%)
Individual background	
Presence of children	
Yes	4,015 (73.79%)
No	1,426 (26.21%)
Living with parents	
Living near or with parents	1,567 (28.80%)
Live separately from parents	3,874 (71.20%)
Parents' occupation	
Father	
Medical professional	1,789 (32.88%)
Not a medical professional	3,390 (67.12%)
Mother	
Medical professional	1,043 (19.17%)
Not a medical professional	4,398 (80.83%)
Parents' total annual income (JPY)	
<10,000,000	1,852 (34.04%)
≥10,000,000	1,746 (32.09%)
Unknown	1,843 (33.87%)
Birthplace	
City	2,029 (37.29%)
Rural	3,412 (62.71%)

Competitiveness scores among physicians

The mean total competitiveness score for the overall sample was 58.79 (SD: 10.52), with a 95% CI of 58.51-59.07. The summary statistics for the Competitiveness Scale are presented in Table 2. The possible range for the total score was 20-140.

**Table 2:** Score of scale of competition The Competitiveness Scale was developed by Sekiguchi [1]. In this study, responses with a total score of <50 were excluded from the analysis; thus, the observed range is 50-140. SD: standard deviation; CI: confidence interval

Variable	Mean±SD	95% CI	Range
Total competitiveness score	58.79±10.52	58.51-59.07	50-140

Competitiveness scores by medical specialty

Unadjusted comparisons revealed significant differences in competitiveness scores across specialties (Table 3). The highest mean scores were observed among junior residents (mean=62.17; SD=10.60), followed by physicians in Plastic Surgery (mean=61.94; SD=9.43) and Emergency Medicine (mean=60.21; SD=10.85). In contrast, the lowest mean scores were found among physicians in Psychiatry (mean=57.03; SD=10.96) and Obstetrics and Gynecology (mean=57.33; SD=10.98).

**Table 3:** Competitiveness scores by medical specialty Departments are listed in ascending order of mean competitiveness scores. "Resident" refers to junior residents undergoing initial postgraduate training. Differences in competitiveness scores across specialties were analyzed using the one-way ANOVA/Kruskal-Wallis test. SD: standard deviation; IQR: interquartile range

Department	n	Competitiveness score (mean±SD)	Median (IQR)
Psychiatry	359	57.03±10.96	57 (51-63)
Obstetrics and Gynecology	138	57.33±10.98	58 (51-65)
General Medicine	105	57.49±10.05	59 (52-64)
Dermatology	116	57.62±11.34	58 (52-66)
Clinical Laboratory Medicine (other)	106	57.95±12.13	60 (52-66)
Otolaryngology, Head and Neck Surgery	136	58.26±10.52	58 (52-66)
Orthopedic Surgery	279	58.40±10.41	59 (53-64)
Radiology	127	58.41±9.57	57 (52-66)
Pediatrics	313	58.46±10.12	60 (53-65)
Rehabilitation Medicine	57	58.65±11.13	61 (52-66)
Anesthesiology	166	58.74±10.44	59.5 (53-65)
Internal Medicine	2,207	58.77±10.60	59 (53-65)
Ophthalmology	123	58.97±10.51	60 (55-68)
Neurological Surgery	144	59.10±10.43	59 (54-66)
Urology	118	59.14±8.55	59.5 (54-64)
Pathology	27	59.22±10.56	59 (53-63)
Surgery	134	59.62±9.94	60 (54-66)
Specific Surgery	452	59.85±10.16	60 (54-66)
Emergency Medicine	77	60.21±10.85	60 (55-67)
Plastic Surgery	49	61.94±9.43	60 (55-68)
Resident	208	62.17±10.60	62 (55-69)

Multivariable analysis for factors associated with competitiveness scores

Tables 4-5 present the results of the multivariable linear regression analysis examining factors associated with competitiveness scores. Regarding medical specialty, after adjusting for potential confounders, junior residents demonstrated significantly higher competitiveness scores compared to physicians of Internal Medicine (reference), with a regression coefficient (β) of 1.65 (95% CI: 0.06-3.24; p=0.042). Conversely, Psychiatry was associated with significantly lower scores (β=-1.67; 95% CI: -2.83 to -0.51; p=0.005). No other specialties showed statistically significant differences compared to Internal Medicine.

**Table 4:** Factors associated with competitiveness scores in multivariable linear regression analysis CI: confidence interval

Variables	Coefficient	95% CI	P-value
Department			
Internal Medicine	0 (reference)		
Surgery	0.02	-1.78 to 1.83	0.979
Specific Surgery	0.53	-0.53 to 1.60	0.325
Psychiatry	-1.67	-2.83 to -0.51	0.005
Neurological Surgery	0.51	-1.24 to 2.26	0.568
Plastic Surgery	2.77	-0.16 to 5.70	0.064
Ophthalmology	-0.02	-1.91 to 1.87	0.982
Pediatrics	-0.39	-1.62 to 0.84	0.531
Obstetrics and Gynecology	-0.97	-2.75 to 0.81	0.284
Dermatology	-1.05	-2.99 to 0.89	0.288
Otolaryngology, Head and Neck Surgery	-0.98	-2.77 to 0.82	0.287
Urology	0.80	-1.84 to 2.00	0.935
Orthopedic Surgery	-0.41	-1.70 to 0.88	0.533
Rehabilitation Medicine	0.37	-2.35 to 3.09	0.789
General Medicine	-1.64	-3.67 to 0.39	0.113
Anesthesiology	-0.14	-1.78 to 1.51	0.871
Radiology	-0.73	-2.59 to 1.13	0.444
Emergency Medicine	0.49	-1.87 to 2.86	0.683
Pathology	-0.83	-4.76 to 3.10	0.678
Clinical Laboratory Medicine (other)	-0.12	-2.17 to 1.93	0.907
Resident	1.65	0.06 to 3.24	0.042

**Table 5:** Factors associated with competitiveness scores in multivariable linear regression analysis CI: confidence interval; JPY: Japanese yen

Variables	Coefficient	95% CI	P-value
Age (continuous)	0.05	0.03 to 0.06	<0.001
Sex			
Female	0 (reference)		
Male	2.37	1.57 to 3.17	<0.001
Job characteristics			
Leadership position			
No	0 (reference)		
Yes	0.37	-0.28 to 1.01	0.268
Engaged in rural healthcare			
No	0 (reference)		
Yes	0.66	-0.06 to 1.38	0.074
Number of beds where they work			
0-19	0 (reference)		
20-99	-0.07	-1.40 to 1.27	0.923
100-299	-0.70	-1.57 to 0.17	0.133
300 or more	1.02	0.24 to 1.81	0.011
Unknown	-0.30	-1.75 to 1.15	0.684
Individual background			
Presence of children			
No	0 (reference)		
Yes	0.98	0.28 to 1.69	0.006
Living with parents			
Live separately from parents	0 (reference)		
Living near or with parents	0.76	0.14 to 1.37	0.016
Parents' occupation			
Father			
Not a medical professional	0 (reference)		
Medical professional	-0.43	-0.11 to 0.24	0.208
Mother			
Not a medical professional	0 (reference)		
Medical professional	-0.15	-0.90 to 0.61	0.704
Parents' total annual income (JPY)			
<10,000,000	0 (reference)		
≥10,000,000	1.11	0.38 to 1.83	0.003
Unknown	-1.27	-1.95 to -0.59	<0.001
Birthplace			
City	0 (reference)		
Rural	-0.13	-0.71 to 0.44	0.646

Demographic and facility factors showed independent associations. Older age (β=0.05; 95% CI: 0.03-0.06; p<0.001) and male gender (β=2.37; 95% CI: 1.57-3.17; p<0.001) were significantly associated with higher competitiveness. Regarding workplace size, physicians working in large hospitals with ≥300 beds had significantly higher scores compared to those in clinics with 0-19 beds (β=1.02; 95% CI: 0.24-1.81; p=0.011).

In terms of socioeconomic and family background, higher competitiveness was associated with having children (β=0.98; 95% CI: 0.28-1.69; p=0.006), living with or near parents (β=0.76; 95% CI: 0.14-1.37; p=0.016), and a higher parental household income of ≥10 million JPY (β=1.11; 95% CI: 0.38-1.83; p=0.003). Leadership position and experience in rural medicine were not significantly associated with competitiveness scores in this model.

## Discussion

This study is the first large-scale nationwide investigation to examine the distribution of competitiveness and its correlates among Japanese physicians across diverse practice settings. Unlike previous studies that have focused on specific subgroups, our findings draw from a broad sample of over 5,000 physicians. The results revealed two key patterns: (1) while statistical differences in total competitiveness scores were observed across specialties, highest among junior residents and lowest among psychiatrists, the absolute magnitude of these differences was relatively modest; and (2) individual factors such as age, gender, workplace size, and socioeconomic background showed independent associations with competitiveness. These findings suggest that competitiveness among Japanese physicians is a multidimensional construct influenced by a complex interplay of career stage, environmental context, and personal background.

Competitiveness across specialties and career stages

A notable finding was significantly higher competitiveness scores among junior residents compared to internal medicine physicians and other established specialists. This likely reflects the intense structural pressures characteristic of the early career phase, such as competition for competitive specialty training programs and high-stakes board certification exams. Early-career competition is strongly and consistently associated with future positioning [17], which aligns with our observations. However, this finding must be interpreted cautiously; while heightened competitiveness drives skill acquisition, it is associated with the risk of burnout [18]. Psychiatrists showed the lowest competitiveness scores. This may be attributable to the specialty's inherent emphasis on empathy, collaboration, and long-term therapeutic relationships rather than procedural speed or acute intervention. Previous research suggests that psychiatrists tend to score higher on agreeableness and lower on competitive traits [19]. This qualitative difference should not be interpreted as a lack of motivation [20]. Given recent reports of increasing interest in psychiatry in Japan [21], further research to understand these dynamics is merited. Crucially, however, the regression coefficients for specialty differences were generally small. This implies that competitiveness may be a widely observed characteristic among physicians, rather than a fixed trait sharply divided by specialty boundaries.

Personal attributes and socioeconomic background

Older physicians tended to have higher competitiveness scores. While this might reflect increased self-efficacy gained through experience [22], a "survivor bias", where highly competitive individuals remain in active practice longer, may be present. Additionally, the nature of competition may evolve from clinical rivalry in youth to organizational leadership in later years. Male physicians demonstrated significantly higher competitiveness than females. We hypothesize that this disparity might be partly influenced by deeply ingrained sociocultural gender norms. Traditional stereotypes that associate competitiveness with masculinity and cooperation with femininity [23] might socially constrain the expression of competitive traits in female physicians [24]. Furthermore, structural barriers, such as the scarcity of female role models in leadership positions, could potentially influence these psychological orientations [25]. Interestingly, socioeconomic factors, specifically higher parental income and co-residence with parents, were associated with higher competitiveness. This suggests a hypothesis that family support systems might provide a safety net encouraging the pursuit of ambitious careers. Economic advantages facilitate access to educational opportunities and career advancement [26,27], a trend that appears to persist into professional medical practice. However, these sociocultural and socioeconomic explanations remain speculative, and future qualitative studies are needed to explore the underlying mechanisms.

Work environment and competitiveness

Physicians working in large hospitals (≥300 beds) had significantly higher competitiveness scores compared to those in smaller clinics. Large academic medical centers typically demand advanced clinical specialization and research productivity, environments that may attract and foster higher competitiveness. In such organizations, competition for academic promotion and leadership roles is often intense [28,29], which likely contributes to the higher scores observed in this group.

Significance and strengths

The primary strength of this study is the use of a large, nationwide dataset (N=5,441), which facilitated a comprehensive analysis of competitiveness correlates with multivariable adjustment. This study provides a foundational baseline for understanding physician motivation in the Japanese context by clarifying the associations between competitiveness and factors like career stage and workplace size.

Limitations

The limitations merit further consideration. First, although the sample was large, the estimated response rate of 2.7% may introduce non-response bias. Furthermore, the a priori exclusion of participants with scores below 50 to ensure data quality might have inadvertently restricted the lower end of the score distribution, leading to selection bias. Second, the reliance on self-reported questionnaires introduces the risk of self-report bias, particularly social desirability bias. Physicians might subconsciously underreport "hypercompetitive" attitudes due to collaborative professional norms. Third, while our multivariable analysis identified several statistically significant factors, these results should be interpreted with caution regarding their clinical and practical significance. Given the large sample size, even minor differences yielded statistical significance. The actual magnitude of these associations, as indicated by the relatively small regression coefficients, is modest. Therefore, these findings should be viewed as broad demographic trends rather than deterministic drivers. Fourth, regarding the variable structure, "Junior Resident" was treated as a distinct category alongside medical specialty. While residents are in a transitional career stage rather than a fixed specialty, this classification was necessary to explicitly compare early-career physicians with established practitioners. Finally, as a cross-sectional study, causal relationships could not be inferred. Future longitudinal research should track how competitiveness evolves as residents mature into specialists.

## Conclusions

This large-scale nationwide survey comprehensively examined the distribution of competitiveness and its associated factors among Japanese physicians. Total competitiveness scores were highest among junior residents and lowest among psychiatrists. While statistically significant distinctions were observed across specialties, the modest effect sizes indicate that competitiveness should not be definitively interpreted as a fixed or stable professional trait. Given the cross-sectional design of this study, competitiveness appears to be a widely observed but dynamic characteristic influenced by career stage, environmental context, and personal background. Furthermore, higher competitiveness was independently associated with male gender, employment at large hospitals (≥300 beds), having children, living with or near parents, and a higher parental household income. These findings provide a vital baseline for future longitudinal research and for designing career support systems that foster both professional growth and well-being.
